# Supplementary material for: The structure of the Tad pilus alignment complex reveals a periplasmic conduit for pilus extension
Source: Nat Commun. 2025 Jul 29;16:6977. doi: 10.1038/s41467-025-62457-8 (PMC12307814; doi:10.1038/s41467-025-62457-8)
Supplement: Supplementary file 2 — Description of Additional Supplementary Files [file 41467_2025_62457_MOESM2_ESM.pdf]

Description of Additional Supplementary Files

Supplementary Data 1: Atomic coordinates for the structural model of the full Tad pilus complex

Supplementary Video 1: Cryo-EM map, and associated atomic model, of the RcpC dodecamer

Supplementary Video 2: Interfaces between RcpC subunits within the dodecameric complex.
